# Considerations for the genotoxicity assessment of middle size peptide drugs containing non-canonical amino acid residues

**DOI:** 10.1186/s41021-023-00294-1

**Published:** 2023-12-13

**Authors:** Masayuki Mishima, Kei-ichi Sugiyama

**Affiliations:** 1 Translational Research Division, Chugai Pharmaceutical Co., Ltd, Yokohama, Kanagawa 244-8602 Japan; 2 Division of Genetics and Mutagenesis, National Institute of Health Sciences, Kawasaki, Kanagawa 210-9501 Japan

**Keywords:** Peptide drugs, Cyclic peptides, Genotoxicity, Impurities, Guidelines

## Abstract

**Background:**

Middle size peptides (MSPs) have emerged as a promising new pharmaceutical modality. We are seeking the best way to assess the non-clinical safety of MSPs.

**Consideration:**

The requirements for assessing the genotoxicity of pharmaceuticals differ between small molecule drugs and biotherapeutics. Genotoxicity tests are necessary for small molecule drugs but not for biotherapeutics. MSPs, however, share similarities with both small molecule drugs and biotherapeutics. Here, we describe important points to consider in assessing the genotoxicity of MSP drugs. The current standard of genotoxicity assessment for small molecules may not be entirely appropriate for MSP drugs. MSP drugs need genotoxicity assessment mostly according to the current standard of small molecule drugs.

**Conclusion:**

We propose a few modifications to the standard test battery of genotoxicity tests, specifically, the inclusion of an in vitro gene mutation test using mammalian cells, and exclusion of (Q)SAR assessment on MSP-related impurities.

## Background

Since the invention of flexizyme, middle size peptides (MSP) containing non-canonical amino acid residues have emerged as a promising new modality that can expand therapeutic targets. Flexizyme enabled the incorporation of non-canonical amino acids into peptides to form artificial molecules with characteristics that would be unattainable using only the 20 canonical human amino acids [1]. Other methods for building peptides out of non-canonical structures have also been reported [2–5]. With these methods, we can create MSPs with novel functions that would be difficult to synthesize with traditional methods [6–10]. Currently, there is much interest in cyclic MSP drugs due to their in vivo stability, membrane permeability, and large surface area for interacting with target molecules.

When a new modality drug appears, we must determine the best way to non-clinically assess its safety during drug development. In the early days of antibody drugs, we stuck to the traditional methods of non-clinical safety evaluation that had been optimized for small molecule drugs. This usually involved genotoxicity tests and in vivo tests using non-reactive animal species. The results of these studies offered little to no useful information on human safety. The traditional approach fails to alert against cytokine release syndrome, which is a known adverse effect of antibody drugs that can be lethal [11]. Investigating the proper way to assess MSPs will make clinical studies safer and eliminate useless experiments.

There is no clear consensus on the defined parameters of an MSP drug. Exactly how big is middle size? Can D-amino acids found at very low levels in human body be called canonical? For this discussion, we define an MSP as having non-canonical and/or D-amino acid residues as components, and a molecular weight of 1,000 or larger.

## Do we need genotoxicity assessment for MSPs?

Peptides and their derivatives fall with the scope of the ICH S6(R1) guideline [12] produced by the International Council for Harmonisation of Technical Requirements for Pharmaceuticals for Human Use (ICH) on the preclinical safety evaluation of biotechnology-derived pharmaceuticals. According to the guideline, “It is not expected that these substances would interact directly with DNA or other chromosomal material, peptides and their derivatives are generally recognized non-genotoxic, and do not need genotoxicity tests.” The ICH S6 guideline was first published in 1997 and was last updated in 2011. At that time, “peptides and their derivatives” probably referred to canonical peptides like natural insulin and modified insulin. Therefore, the ICH S6(R1) guideline would not be applicable to current MSPs.

MSP drugs can permeabilize the cell membrane. Cyclosporine (CASRN 59865–13-3, MW = 1,203) produced by *Tolycocladium*, composed of 11 amino acid residues, 8 of which are non-canonical, is often referred to as a representative example of an MSP. Cyclosporine is orally absorbed and binds to cyclophilins inside T cells to cause immune suppression [13]. Flexible conformation changes, cell-penetrating peptide motifs, aromatic amino acids, and basic residues help give MSPs membrane permeability [14, 15]. Many researchers are aiming to apply MSPs to intracellular drug targets. We therefore need to assess their genotoxicity.

**Fig. 1:**
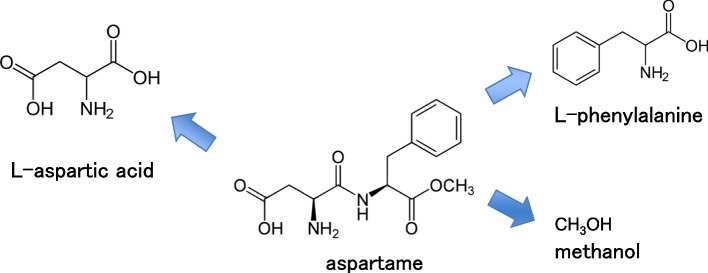
Aspartame and metabolites

We recommend genotoxicity assessment for every MSP, although some may not permeabilize the cell membrane. Even if an MSP itself has no membrane permeability, its metabolites or degradation products can enter the cells. Aspartame (CASRN 22839-47-0, Fig. 1) is an example of simple non-canonical peptide composed of non-canonical methyl L-phenylalanine and L-aspartic acid. Aspartame is hydrolyzed or metabolized into methanol, L-aspartic acid, and L-phenylalanine [16]. Low-molecular-weight metabolites can enter cells and may interact with intracellular components.

## Application of ICH S2(R1)

ICH S2(R1) describes the consensus on methods for appropriately evaluating the genotoxicity of small molecular drugs [17]. S2(R1) recommends two options for such assessment. The great majority of pharmaceutical companies choose the first option in their development programs. This includes the following test battery:

i. A test for gene mutation in bacteria (Ames test).
ii. A cytogenetic test for chromosomal damage (the in vitro metaphase chromosome aberration test or in vitro micronucleus test), or an in vitro mouse lymphoma Tk gene mutation assay.
iii. An in vivo test for genotoxicity, generally a test for chromosomal damage using rodent hematopoietic cells, either for micronuclei or for chromosomal aberrations in metaphase cells.

**Fig. 2:**
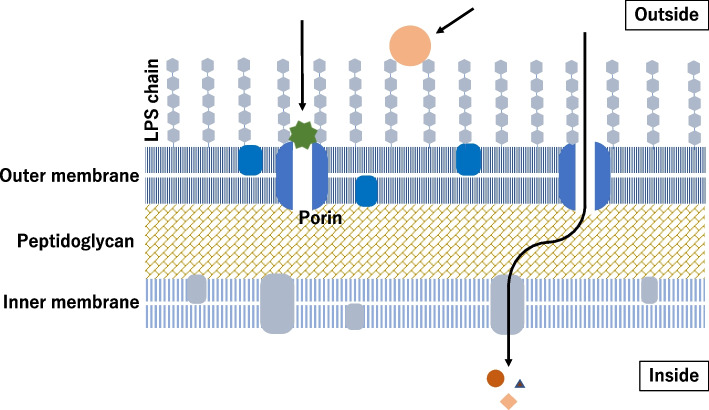
Gram negative bacterial membrane

We think that this option needs to be slightly modified for MSPs. It is questionable whether the Ames test is suitable for MSPs. Ames tester strains are series of *Salmonella typhimurium,* a gram negative bacteria whose outer membrane is equipped with lipopolysaccharide (LPS) chains (Fig. 2). The outer membrane is a strong permeability barrier against foreign substances. An external material must penetrate both the LPS layer and outer membrane porins, which are passive diffusion channels that prevent larger molecules from permeating [18]. *S.typhimurium* Ames tester strains have *rfa* mutations that allow for better permeability of the outer membrane, which was examined for sensitivity to crystal violet (CASRN 548–62-9, MW = 408) [19]. The size limit of molecules able to permeate the outer membrane has not been clarified.

We question the compatibility of the Ames test on MSP and recommend a gene mutation assay with mammalian cells in addition to the Ames test. Various *rfa* mutations enhanced sensitivity to antibacterial agents comparing with that of wild type *S typhimurium* [20]. For low molecular antibiotics, *rfa* mutations effectively enhanced permeability. Relative minimum inhibition concentration (rMIC) against novobiocin (CASRN 303–81-1, MW = 613) was 0.05 or lower with *rfa(R-res-2)*, *rfaG* or *rfaF* mutant strains. For larger molecules, *rfa* mutations were not necessarily effective. rMIC against a glycopeptide antibiotic, vancomycin (CASRN 1404–90-6, MW = 1449), was 0.3 with *rfaF*, 0.4 with *rfaG* or 1.0—1.1 with *rfa(R-res-2)* mutants. rMIC to a cyclic peptide antibiotic, polymixin B (CASRN 1405–26-8, MW = 1189) was 0.1 with all the three mutants. Some *rfa* mutations may not sufficiently enhance permeability of MSP.

Bleomycin (CASRN 11056–06-7, MW = 1416) is a potent mutagen in mammalian cells in HPRT assay with CHO and V79 cells [21] and in TK assay with mouse lymphoma L5178Y cells [22]. Bleomycin mainly binds to -GC- or -GT- dinucleotides and causes DNA strand breaks [23, 24]. Bleomycin was negative in Ames test with TA98 or TA100 tester strains [25] with *rfa* mutation and the target hot spot -GCGCGCGC- 8 repetitive -GC- residues or -CCC- site [19], suggesting that Ames test might not be useful for MSP larger than MW = 1400. Bleomycin was positive with TA102 that is highly sensitive to oxidative stress [21]. We did not consider that the positive result with TA102 supported permeability of bleomycin through the outer membrane because bleomycin produced hydrogen peroxide [23] that could induce positive response in TA102.

We still recommend performing the Ames test when a test article is larger than MW = 1,400. Because details of the *rfa* mutations of Ames tester strains have not been elucidated, the range of Ames effective MSPs could not be clarified. Even if MSP cannot penetrate the bacterial outer membrane, its metabolites may be able to enter and affect DNA. In the future, after accumulating more data on MSPs, we can establish a more appropriate strategy for assessing genotoxicity.

Given the above considerations, a current example of a recommended test battery for MSPs is:

i. an Ames test.
ii. an in vitro mouse lymphoma Tk gene mutation assay, or a cytogenetic test for chromosomal damage (in vitro metaphase chromosome aberration test or in vitro micronucleus test) plus an in vitro mammalian cell gene mutation assay other than a Tk assay, e.g. HPRT assay.
iii. An in vivo test for genotoxicity, generally a test for chromosomal damage using rodent hematopoietic cells, either for micronuclei or for chromosomal aberrations in metaphase cells.

The proposed test battery should be reconsidered after accumulating more data.

## Application of ICH M7(R1)

An assessment of the mutagenicity of impurities is needed because mutagenic chemicals such as alkylating agents are often used in the synthesis of MSP. The M7(R1) guideline is useful for small molecule synthetic impurities, e.g. starting materials, reagents and residual solvents [26].

**Fig. 3:**
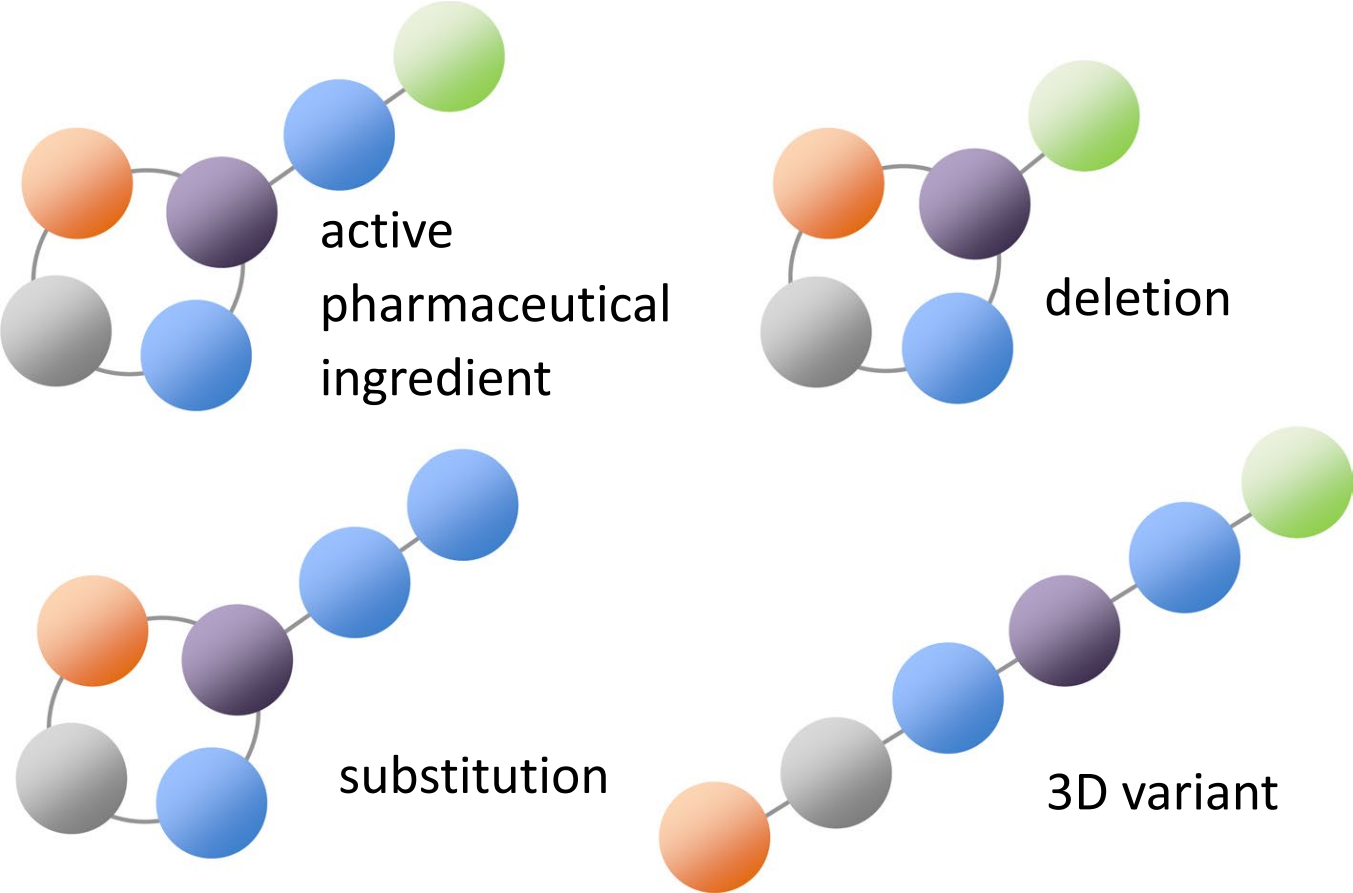
MSP-related impurities

We however think that this guideline is also unapplicable to MSP-related impurities (Fig. 3). M7(R1) requires the assessment of mutagenicity for all potential impurities. A vast number of assessments have been enabled by in silico quantitative structure activity relationship (QSAR) tools. Currently available QSAR tools were established based on accumulated Ames test data from the public domain and industry databases, but these sources contain very little data on middle or large size molecules like MSPs. The MSP itself and MSP-related impurities do not reside in the chemical spaces where QSAR tools are effective. Additionally, we are afraid that the estimated Ames results on MSPs may not be entirely relevant to human safety for the reasons described in the previous section (Application of ICH S2(R1)).

**Fig. 4:**
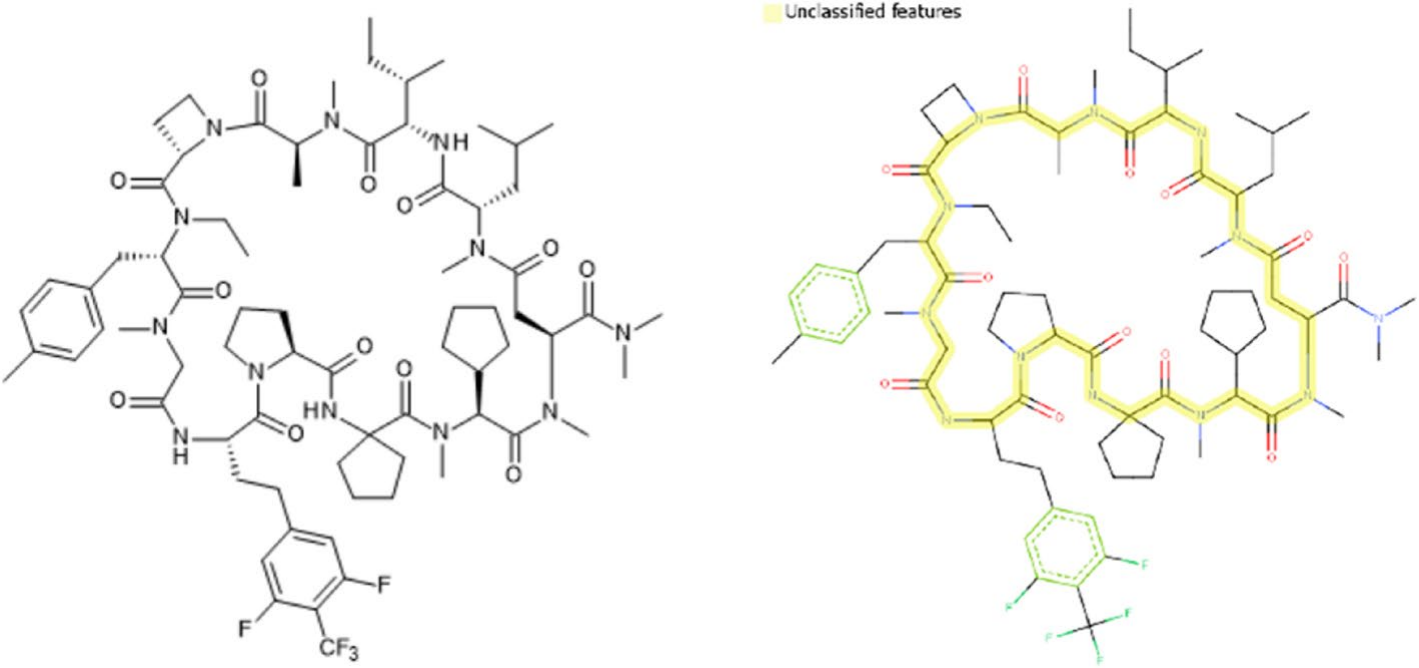
A MPS example, LUNA18, containing features that were not found in the Lhasa Ames test reference set of Derek Nexus 6.0.1

**Fig. 5:**
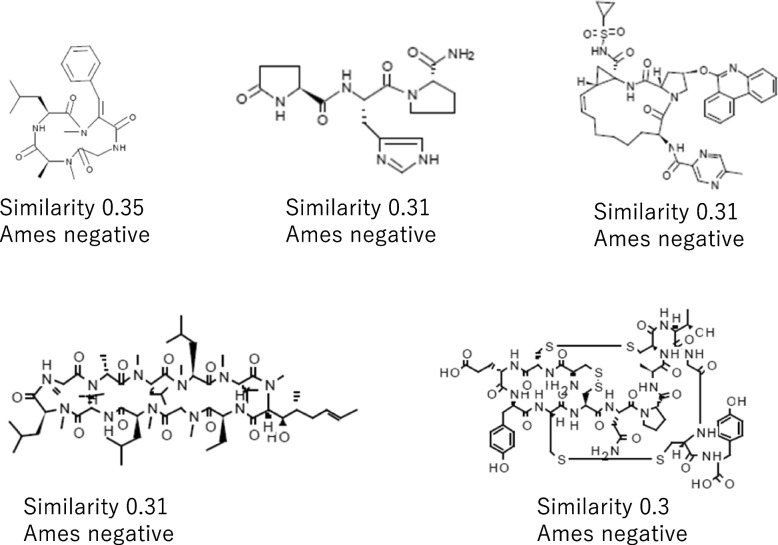
Analog structures of LINA-18 from training/reference set of Leadscope Model Applier v2.4.1–36

An example of QSAR assessment on a MSP drug candidate under development, LUNA18 [27], was shown in Figs. 4 and 5. No mutagenicity alert came out from either a knowledge-base or a statistic-base QSAR tool. The backbone structure of LUNA18 was out of chemical space of the Derek reference set (Fig. 4). Although five analog chemicals were found in the training/reference set of Leadscope Model Applier, their similarity scores were only 0.35 or less (Fig. 5).

Because QSAR is not applicable to MSP-related impurities, we cannot—therefore do not need to—assess them according to ICH M7(R1). In case an MSP-related impurity is > 1 mg/day where genotoxicity evaluation is recommended in the context of ICH M7(R2), we recommend conducting in vitro evaluation using genotoxicity tests according to the modified version of the ICH S2(R1) guidance that we describe in the previous section.

## Conclusions

- MSPs need special consideration for genotoxicity assessment due to the probable inapplicability of the Ames test.
- We recommend the inclusion of an in vitro mammalian cell gene mutation assay to complement the Ames test.
- QSAR assessment is needed for small molecule impurities but not for MSP-related impurities.
- For an MSP-related impurity > 1 mg/day, Ames test, in vitro mammalian gene mutation assay, and in vitro chromosomal aberration or micronucleus assay, are recommended.
- The above recommendations should be reconsidered after the accumulation of data on MSP.

## Data Availability

Not applicable.
